# Assessing the impacts of COVID-19 on Care Homes in Wales.

**DOI:** 10.23889/ijpds.v7i3.1985

**Published:** 2022-08-25

**Authors:** Richard Fry, Joe Hollinghurst, Laura North, Chris Emmerson, Sara Long, Ashley Akbari, Mike Gravenor, Ronan Lyons

**Affiliations:** 1 Swansea University; 2 Public Health Wales; 3 Aneurin Bevan University Health Board; 4 Health Data Research UK - Wales and Northern Ireland, Swansea University

## Objectives

A defining feature of the COVID-19 pandemic in many countries were the tragic extent to which care home residents were affected and the difficulty in preventing the introduction and subsequent spread of infection.

## Approach

Utilising linked data in the SAIL Databank we set out to develop a linked data platform as part of the ‘One Wales’ approach to generate evidence to inform policy makers on the key areas of transmission pathways, care home characteristics, excess mortality, and the impacts of vaccination. We used multi-sectoral linked data including routinely collected health data, administrative data and GIS generated metrics on care home characteristics and community infection rates to better understand how multiple factors impacted on care home residents.

## Results

We created a care home index with enhanced care home characteristics for all care homes in Wales and were able to link 15,773 care home residents in the SAIL Databank to 923 care homes. We were able to generate early evidence demonstrating an increased risk of mortality for care home residents during Wave 1 (adjusted HR 1.72 compared to 2016). We were able to show that hospital discharge in Wales during the initial stages of the pandemic, although significant, had a much smaller impact on subsequent infections than care home size and accounted for 1.8% of infected discharge events. We also showed that community prevalence, inpatient appointments and people living with dementia all contributed to increased risks of catching COVID in a care home.

## Conclusion

The response of the ‘One Wales’ team and the SAIL linked data platform facilitated meaningful insight on the impacts of COVID in social care settings in Wales. The evidence generated was used by policy makers from Welsh and UK Governments to inform policy direction as the pandemic progressed.

